# Revealing the Inner Changes of Component Composition Derived from DOM PARAFAC Based on Two-Dimensional Correlation Spectroscopy

**DOI:** 10.3390/molecules27217316

**Published:** 2022-10-27

**Authors:** Hongyang Cui, Lina Xie, Guogang Zhang, Yue Zhao, Zimin Wei

**Affiliations:** 1 Tianjin Key Laboratory of Animal and Plant Resistance, College of Life Sciences, Tianjin Normal University, Tianjin 300387, China; 2 College of Life Science, Northeast Agricultural University, Harbin 150030, China; 3 Laboratory for Earth Surface Processes, College of Urban and Environmental Sciences, Peking University, Beijing 100871, China

**Keywords:** compost, DOM dynamic, excitation-emission matrices (EEMs), multidimensional data analysis, two-dimensional correlation spectroscopy (2DCOS)

## Abstract

Plenty of humic acid components compositions are contained in dissolved organic matter (DOM) derived from composting. Fluorescence signals were employed to characterize the changes in DOM components in the component process. In the composting process, five individual DOM fluorescence parallel factor analysis (PARAFAC) components were identified. At the end of the composting, PARAFAC component C5, which represented high humification and complex structure compounds, was detected, but the simple structure DOM PARAFAC component C1 was absent. In this study, a technique combining EEM-PARAFAC with two-dimensional correlation spectroscopy (2DCOS) further supplied detailed information about the dynamics of DOM peaks in PARAFAC components. 2DCOS results showed that the variation of the peaks in PARAFAC components was different in the composting process. The formation of a complex DOM fluorescence substance was attributed to the residues from the simple fluorescence peak degradation. The evolution of the DOM fluorescence peaks in each PARAFAC component indicated that simple structure compounds helped the formation of the complex DOM fluorescence substance in the composting process. These results revealed that EEM/PARAFAC combined with 2DCOS could be used to track the evolution of DOM PARAFAC components during the composting process.

## 1. Introduction

Composting is a prevalent and appropriate waste treatment method. It is a cheap, effective, and sustainable way to manage organic solid waste [1,2]. During composting, the organic solid wastes are transformed into complex substances by microorganisms in the aqueous phase [3]. In the process of composting, dissolved organic matter (DOM) is considered the most active portion in—and is frequently employed to evaluate the quality of—composts. Thus, depicting the dynamic of DOM molecules is important for understanding the dynamics of the organic matter and obtaining exact information on the relationships among the fluorescence peaks in DOM molecules.

Composting-originated DOM contains complex substances of humification and degradation [4]. On the other side, the characteristics of DOM significantly change as the composting process progresses [5,6]. Consequently, it is difficult for us to obtain accurate information about the dynamic of DOM molecules in the composting process.

Incorporating excitation-emission matrices (EEMs) with parallel factor analysis (PARAFAC) is a significant technique for DOM characterization. Based on this technique, individual molecules can be decomposed from DOM EEMs. This method has been positively employed to assess the environmental dynamics of DOM in diverse natural ecosystems [7,8], fluorescent DOM fractions in landfill leachates [9], and the properties and behaviors of composting-derived DOM [10]. Researchers use maximum fluorescence intensities (Fmax) or the relative contribution of PARAFAC molecules to describe the variation in the specific molecules [10,11,12]. However, generally, two or more fluorescence peaks existed in PARAFAC molecules [6,13,14]. Therefore, detailed internal peaks cannot be explained by Fmax or a value representing the overall changes in a DOM molecule. Consequently, to depict the biological process of DOM during composting, the evolution of DOM fluorescence peaks needs to be further investigated.

Generalized two-dimensional correlation spectroscopy (2DCOS) is considered a spectrum-valid investigative method, which can offer significant help and benefit to various spectroscopic investigations [15,16]. The features of generalized 2DCOS are: (1) improvement in the resolution of spectra, (2) founding of definite assignments using the relationship of bands selectively joined with several interaction dynamics, and (3) revealing the spectral peak emergence sequence. In particular, the hetero-2DCOS can compare two sets of spectra under different probes from the same sample under the same perturbation. The continuous variables can be employed as the perturbation conditions during the evolution of DOM during composting and the concentration of contaminants in the process of binding onto DOM [17,18,19]. Owing to the data-analytical capability of 2DCOS, composting time was employed as the external perturbation to explore the inner changes of DOM molecules. Based on treating the response patterns generated by the two different molecules during composting as a perturbation, the relationships between the signals from these two spectra can be detected. Therefore, 2DCOS combined with PARAFAC can be employed to describe the dynamic of DOM during composting.

In this work, 2DCOS combined with EEM-PARAFAC was used to provide an in-depth characterization of the dynamics of DOM peaks in PARAFAC molecules in the composting process. This study aims to illustrate the dynamic of compost-derived DOM in the composting process.

## 2. Results and Discussion

### 2.1. DOM Characterization Using PARAFAC Analysis

**Figure 1:**
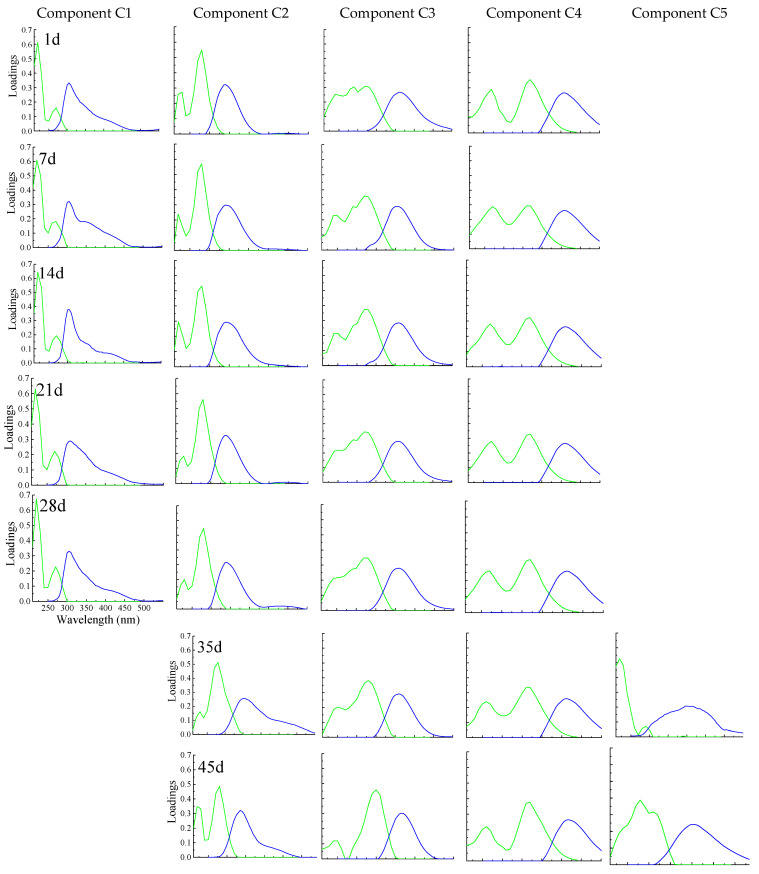

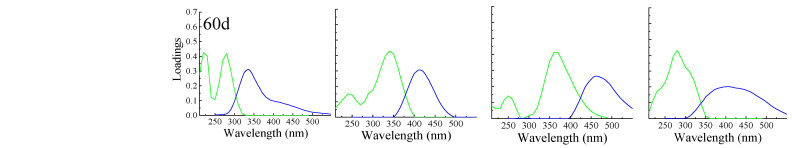
DOM PARAFAC components on composting days 1, 7, 14, 21, 28, 35, 45, and 60. The excitation wavelengths of the DOM components were plotted with blue lines, and the emission wavelengths of the DOM components were plotted with green lines. From composting days 1 to 28, components C1 to C4 were identified. From composting days 35 to 60, component C1 vanished with the observation of component C5.

The DOM EEM spectra were analyzed by PARAFAC analysis. Detailed PARAFAC analysis is offered in Text S2. Four fluorescence molecules were found in each sample using EEM spectra combined with PARAFAC analysis (Figure 1). The excitation and emission loadings of the molecules are shown in Figure 1. The EEM spectra of the fluorescent molecules are shown in Figure S3. Four molecules were successfully developed using PARAFAC in each sampling time. The PARAFAC molecules named C1, C2, C3, and C4 were identified at composting times from 1 to 28 d). The PARAFAC molecules named C2, C3, C4, and C5 were identified at composting times from 35 to 60 d. Table 1 offers the features of DOM PARAFAC molecules and provides comparisons with the identified molecules in various natural environments.

Each component of a different sampling time generally possesses more than one DOM fluorescence peak (Figure 1 and Figure S3). In addition, component C1 was not detected after 28 d; simultaneously, component C5 was present, with different excitation emission characteristics from other molecules. The peak positions of PARAFAC molecules C1-5 showed no obvious change (less than 5 nm changes in the wavelength of excitation and emission) during composting. However, the DOM peak intensities in PARAFAC molecules presented changed significantly during composting (Table S2). Additionally, detailed DOM molecule information is provided in Text S3.

### 2.2. Distribution of PARAFAC Molecules during Composting Process

**Figure 2:**
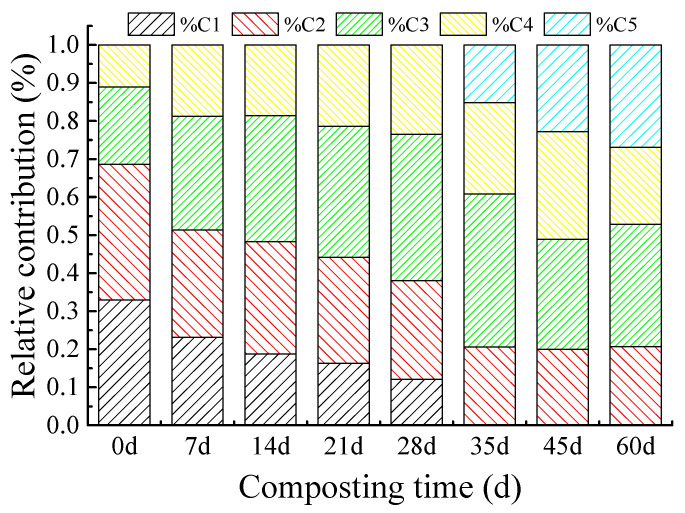
The relative concentration changes in identified DOM PARAFAC components on composting days 1, 7, 14, 21, 28, 35, 45, and 60.

DOM component quantitative information could be provided by PARAFAC analysis [5,28,29,30]. There are no obvious differences for the same PARAFAC component, originating from various composting materials at the same composting time (Figure S4). The average relative contribution of the PARAFAC molecules at each composting time is shown in Figure 2. In the composting process, the average relative contribution (representing the signals of the identified DOM PARAFAC components) of the tyrosine-like component C1 decreased from 32.9% to 0%, and that of the tryptophan-like component C2 steadily decreased from 35.7% to 20.7% (Figure 2), which indicates that tyrosine- and tryptophan-like molecules are decomposed by microbes. The average relative contributions of DOM PARAFAC components C3 and C4 show an increasing trend during composting: component C3 increased from 20.3% to 32.1%, and component C4 increased from 11.1% to 20.3% (Figure 2). These results suggest that these two molecules may accumulate during composting. Component C5 was detected after 28 d and also presented an increasing trend: the average relative contribution of component C5 increased from 0% at the beginning of composting to 26.9% in the final composting stage. We can infer the changes in the molecules during composting based on the relative contribution of each fluorescent component, but we do not know the inner PARAFAC component changes. During composting, the degradation of simple DOM compounds (such as sugars, amino acids, and simple humic substances) directly promotes complex structure humic-like compounds. Therefore, some compounds like components C3–5 accumulated during composting.

### 2.3. Characterization of the Composting-Derived DOM Dynamics Using the Excitation Loadings of PARAFAC Molecules Coupled with 2DCOS

**Figure 3:**
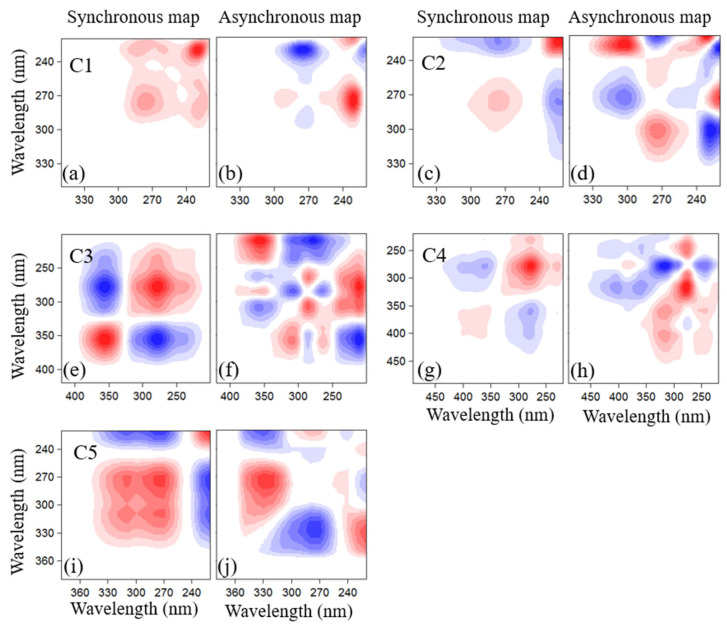
Synchronous and asynchronous 2DCOS analysis characterized by excitation loadings data originating from each DOM PARAFAC component. (Note: Positive and negative cross-peaks were presented in red and blue.) Synchronous maps for components C1 to C5 were presented in Figure 3a,c,e,g,i. Asynchronous maps for components C1 to C5 were presented in Figure 3b,d,f,h,j.

In order to further explore the fate of DOM during composting, the 2DCOS analysis was used to investigate the dynamic of DOM fluorescence peaks. Synchronous and asynchronous 2DCOS built on time-dependent DOM molecules are plotted in Figure 3.

The asynchronous spectra (Figure 3) are antisymmetric with respect to the diagonal line. Based on Noda’s rules, the sequential changing order was expressed by the cross peaks [15]. Thus, the asynchronous spectra exhibit changes when the original bands are out of phase from each other, with one band changing ahead of or behind the other bands. Furthermore, in asynchronous 2DCOS, only cross-peaks can be identified. The same signs for spectral coordinates (λ1, λ2) in both the synchronous and asynchronous maps indicate that the change in the spectral intensity at the λ1 band occurs prior to that at λ2 along the perturbation variable axis. This order is reversed when the signs are opposite.

In the synchronous map of component C1, two auto-peaks were detected at the wavelength pairs of 220/270 nm and are positively correlated with each other (Figure 3a). This result indicates that simple structure DOM component peak B1(C1) and peak B2(C1) varied in the same direction during composting [15,17]. In the component C1 2D asynchronous map (Figure 3b), a positive peak was detected at the wavelength 222/270 nm, and a negative peak was observed at the wavelength pairs of 222/231 nm during composting. Based on Noda’s rule, [15] the variation of the band sequence during composting follows peak B2(C1)→peak B1(C1). This result indicates that peak B2(C1) in component C1 presents a more easily biodegradable property during composting. Particularly interesting is that DOM fluorescence peak at 220 nm in the synchronous-2DCOS was resolved into two bands at 222 nm and 231 nm in the asynchronous 2DCOS, which was not detected in the original spectra. They correspond to a tyrosine-like component at a lower wavelength [8]. In addition, all peaks in component C1 were decomposed (Figure 2), which may be attributed to the easily biodegradable characteristic of this fluorescent substance. During biodegradation, based on the concentration change of the component, peak B1(C1) and peak B2(C1) in component C1 all presented a decreasing trend. These results were in good agreement with the relative quantity change in fluorescence component C1 (Figure 2) and with previous studies about the change in the tyrosine-like component during composting [1,5,31].

Figure 3c,d show the synchronous and asynchronous maps, which were generated from the excitation loadings of fluorescence component C2. Two main auto-peaks (230 nm (peak T1(C2)) and 280 nm (peak T2(C2))) are observed in the synchronous 2DCOS map along with negative cross-peaks, suggesting the different direction of these two bands [15]. In the asynchronous map, the band at 280 nm was resolved into two bands at 275 and 300 nm, which were not detected in the original 1D spectra. They correspond to the tryptophan-like component with higher complex structure (peak T2(C2)) [11,21,22,32]. The band at 230nm was assigned to the tryptophan-like component fluorescence peak T1(C2) [11,21,22,32]. The signs of the cross-peaks in the asynchronous spectra showed that in component C2 the changing sequence of the peaks during composting followed the order of peak T1(C2)→peak T2(C2). Moreover, the relative distribution of molecular C2 presented a steadily decreasing trend (Figure 2). However, peak T1(C2) in component C2 actually had an obvious decreasing trend, whereas peak T2(C2) presented an increasing trend during composting. This result was not in agreement with previous studies about the change of the tryptophan-like component during composting [1,4,10].

The dynamic of DOM PARAFAC component C3 during the composting was also explored, and the synchronous/asynchronous 2DCOS maps are plotted in Figure 3e and f, respectively. Two auto-peaks were detected in the synchronous spectra, which correspond to fluorescence peak M1(C3) and peak M2(C3). However, the change of the fluorescence peak at peak A1(C3) (245 nm) was not detected in the synchronous map. These results indicate that the fluorescence peaks M1(C3) and M2(C3) varied in different directions, although fluorescence peak A1(C3) presented no significant change during composting. Combined with the results of the Excitation loadings (Figure 1) and the relative change of fluorescence component C3 (Figure 2), a result could be inferred that the peak M1(C3) decreased, but peak M2(C3) increased during composting. The signs of the cross-peaks in the asynchronous map indicated that the peak changing sequence during composting follows the order of peak M1(C3)→peak M2(C3) in component C3.

The 2DCOS analysis on excitation loadings of PARAFAC component C4 was investigated, and the synchronous/asynchronous maps were plotted in Figure 3g,h. Auto-peaks were observed at 270 and 370 nm in the synchronous spectra, corresponding to complex structure humic compounds peak A2(C4) and peak C(C4), and a negative cross-peak was detected, suggesting the different directions of these bands’ intensity changes. Based on the results above, peak A2(C4) decreased, but peak C(C4) increased during composting. In asynchronous maps, the cross-peaks showed that in component C4 the changing sequence of the DOM peaks during composting was peak A2(C4)→peak C(C4).

Figure 3i,j show the synchronous and asynchronous 2DCOS spectra in excitation loadings, which were constructed from the time-dependent EEM-PARAFAC component C5 during composting. In the synchronous 2DCOS, negative signs were observed between peak L1(C5) and peak L2(C5), indicating that these two peaks changed in different directions during composting. The asynchronous spectrum shows that the sequence variation of the peaks in component C5 is peak L1(C5)→peak L2(C5).

These results indicate that the characterization of the dynamic of DOM during composting using relative concentration only, which was expressed by Fmax% of PARAFAC molecules, was not an accurate method. Although Fmax% of component C2 presented a decreased trend during composting, the peak T2(C2) was detected with a rising tendency in the 2DCOS analysis. As for components C3 and C4, Fmax% of these two molecules showed an increasing trend during composting, but the results of 2DCOS analysis indicated that only peak M2(C3) in component C3 and peak C(C4) in component C4 were synthesized. Therefore, previous studies that characterized DOM using Fmax% of the fluorescence molecules only could not characterize the inner variations of the PARAFAC component [11,33].

### 2.4. Characterization of the Composting-Derived DOM Dynamics Using the Excitation Spectra of PARAFAC Molecules Coupled with Hetero-2DCOS

To confirm the evolution of the DOM fluorescence peaks in the molecules during composting, a hetero-2DCOS analytical technique was also performed (generated between each PARAFAC component). The positive cross peaks in the synchronous hetero-2DCOS indicated that these two bands had the same origin, but the negative cross peaks indicated that these two bands had different origins [15,16]. Detailed hetero-2DCOS analyses of the fluorescence molecules are presented in the supplementary information (See Text S4 in supplementary information).

Based on the above results, during composting, the substances are decomposed in the following order: peak M1(C3)→B2(C1)→B1(C1)→A2(C4)→ T1(C2)→L1(C5). This result is inconsistent with previous studies, which conclude that fresh organic matter (protein-like component) is decomposed along with the formation of a humic-like component during composting [34]. The substances are formed during composting in the following order: peak M2(C3)→C(C4)→T2(C2)→L2(C5).

### 2.5. Change Speed of the Fluorescence Peaks in Different Composting Materials

**Figure 4:**
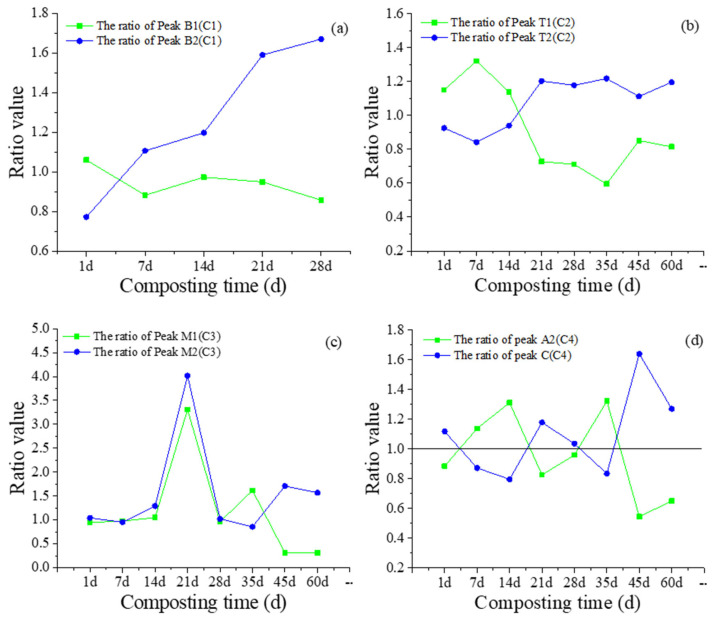
Changes in the ratio of DOM peaks (peak in first group/peak in second group) of PARAFAC molecules during composting. The changes of fluorescence peaks in DOM component C1 (**a**); The changes of fluorescence peaks in DOM component C2 (**b**); The changes of fluorescence peaks in DOM component C3 (**c**); The changes of fluorescence peaks in DOM component C4 (**d**).

The weed, straw, litter, and fruit and vegetable composts were regarded as the same group (first group) because of the high content of cellulose in these raw materials [35]. The chicken manure, swine manure, and food waste composts were considered the same group (second group) because of the high content of protein molecules and similar chemical properties of these raw materials [35]. For this study, the change speed of the fluorescence peaks during composting was tracked by using the ratios of fluorescence intensities of peak X/X’ (e.g., peak B1(C1)/B1(C1)’ represented for peak B1(C1) in first group/ peak B1(C1) in the second group) in the PARAFAC molecules, as plotted in Figure 4. For the ratio of fluorescence peak B1/B1′ in PARAFAC component C1, the ratio value presented a decreased trend during composting (Figure 4a). The ratio of fluorescence peak B2/B2′ in PARAFAC component C1 showed a significantly increasing trend during composting (Figure 4a). These results indicated that in the composting of the first group, fluorescence peak B1(C1) of PARAFAC component C1 was more rapidly decomposed than that in the second group. The degradation rate of fluorescence peak B2(C1) in the composting of the second group was higher than that in the first group. The difference in degradation rate of fluorescence peak B1(C1) and B2(C1) in different groups of composting may be attributed to the different compositions of raw materials, which caused the difference in the amount of tyrosine-like component degradation microbes and the rate of degradation [9,36]. Furthermore, the slope of the ratio of fluorescence peak B1(C1)/B1(C1)’ was significantly higher than that of peak B2(C1)/B2(C1)’ during composting. This result further confirms that the concentration of an easily-degradable component, which can be quickly used by microbes, is higher in the second group of composts.

The ratio of fluorescence peak T1(C2)/T1(C2)’ in PARAFAC component C2 showed a decreased trend during composting (Figure 4b). However, the ratio of fluorescence peak T2(C1)/T2(C2)’ in PARAFAC component C2 presented an increasing trend. This result revealed that in the first group of composts, fluorescence peak T1(C2) of PARAFAC component C2 was more rapidly decomposed than that in the second group of composts, but the decomposition speed of fluorescence peak T2(C2) in the second group was higher than that in the first group of composts.

During the process of 1–28 d, the ratios of peak M1(C3)/M1(C3)’ and M2(C3)/M2(C3)’ in PARAFAC component C3 showed identical trends. After 28 d, the changes in the ratio of peak M1(C3)/M1(C3)’ and M2(C3)/M2(C3)’ presented an inverse trend (Figure 4c). This result indicated that 28 d was a turning point for the decomposition speed of peak M1(C3) and peak M2(C3) during composting. Interestingly, the PARAFAC analysis (Figure 1) showed that component C5 was detected by PARAFAC analysis after 28 d, and component C1 was absent after 28 d, which suggested that an unstable substance was decomposed into a low level, and the concentration of a stable substance increased to a high value.

As shown in Figure 4d, the ratio of peak A2(C4)/A2(C4)’ and ratio of peak C(C4)/C(C4)’ in PARAFAC component C4 presented unstable changes during composting. Interestingly, the changes in the ratio of these two peaks showed an inverse trend during composting, which was antisymmetric with respect to the line of ratio value 1, indicating that change in speed of peak A2(C4) and peak C(C4) in the first group of composts was opposite to that in the second group. This may be attributed to the complex component structures and large component weight of this component, which caused the decomposition speed of peak A2(C4) and formation speed of peak C(C4) in component C4. Therefore, these two peaks presented fluctuation changes during the composting of these two groups.

### 2.6. Dynamics of DOM Molecules during Composting

**Figure 5:**
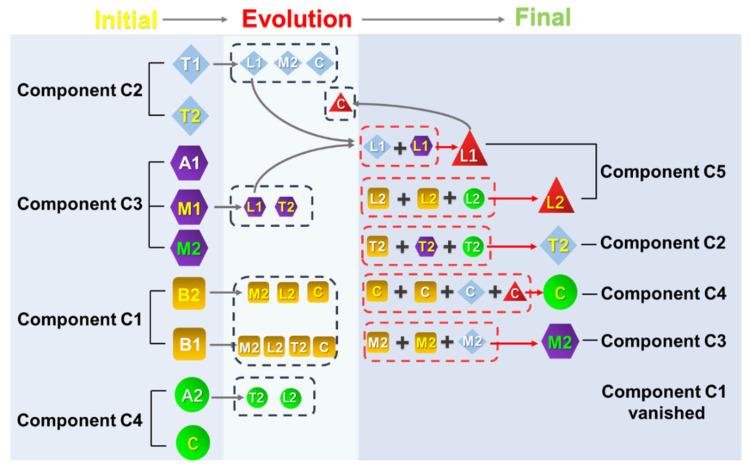
The dynamic of fluorescence molecules during composting was proposed by analyzing the 2DCOS and hetero-2DCOS among the excitation loadings of molecules.

The fate of DOM molecules could be determined based on the 2DCOS maps (Figure 3), which were investigated by excitation loadings of PARAFAC molecules. Summarizing the results of 2DCOS and the variation of fluorescence molecules, the fate of each component during composting was plotted in Figure 5. Peak B1(C1) and peak B2(C1) in component C1 were all degraded during composting (Figure 2 and Figure 5). Peak B1(C1) resolved into four parts, which made a contribution to the formation of Peak T2(C2), peak M2(C3), peak C(C4), and peak L2(C5). Peak B2(C1) was divided into three parts by the activities of microorganisms (Figure 5), which could help to synthesize peak M2(C3), peak C(C4), and peak L2(C5).

The relative concentration of component C2 presented a decreasing trend during composting (Figure 2). However, based on the results of the 2DCOS (Figure 3 and Figure S5), not all the DOM peaks in component C2 were decomposed during composting. Peak T1(C2) was decomposed into three parts during composting, and these parts made a contribution to the formation of peak M2(C3), peak C(C4), and peak L1(C5) (Figure 5). Peak T2(C2) presented a slightly increasing trend. However, the reduction amount of peak T1(C2) was higher than the formation of peak T2(C2). Therefore, the relative concentration of component C2 showed a decreasing trend in the composting process, which is consistent with the relative concentration change of component C2 in PARAFAC.

The change in relative concentration of component C3 during composting showed an increasing trend (Figure 2). However, based on the results of the 2DCOS, not every peak in component C3 was formed (Figure 3 and Figure S5). Peak A1(C3) presented no significant change. Peak M1(C3) was decomposed into two parts, which contributed to the formation of peak T2(C2) and peak L1(C5). Peak M2(C3) presented a significant rising trend (Figure 5).

Component C4 showed an increasing trend during the composting process (Figure 2). However, based on the results of 2DCOS (Figure 3 and Figure S5), peak A2(C4) was decomposed, and peak C(C4) was formed. The residues of peak A2(C4) could help the formation of peak T2(C2) and peak L2(C5) (Figure 5).

The relative concentration of component C5 presented an increasing trend during composting. However, based on the results of the 2DCOS (Figure 3 and Figure S5), the microbial humic fluorescence peak L1(C5), whose chemical property is unstable [26,37], was decomposed after its formation. Peak L1(C5) was transformed into peak C(C4). Peak L2 was related to the microbial-formed fulvic-acid-like substance, whose chemical property is relatively stable [9,24]. Therefore, peak L2(C5) accumulated during composting.

## 3. Experimental Section

### 3.1. Composting Procedure

The composting experiments were conducted at Songjiang Composting Plant in Shanghai, China. A total of seven composting piles were conducted, and each composting pile contained all the materials (vegetables, litter, fruit, weeds, straw, food waste, swine manure, and chicken manure), with the amount of each material approximately equal in each composting pile. Wood shavings and urea were employed as the filler to adjust the C/N of these raw materials to a range of 20–30. After composting was finished, six sampling sites were randomly selected in each composting pile for the sample collection. About 2 t of raw material were contained in each composting pile. To accelerate the composting fermentation, the piles were turned when needed. When the composting piles’ temperature decreased to an ambient temperature at a constant level, the composting was mature. The composting was conducted in autumn, with an ambient temperature of approximately 25 °C. The composting samples were collected on days 1, 7, 14, 21, 28, 35, 45, and 60 from each pile in the composting plant to explore the evolution of the DOM molecules. Six sampling points were selected in each composting pile at each sampling time to analyze DOM. The sampling points were evenly distributed in the composting piles.

### 3.2. Extraction of DOM

Six sampling points were selected, which were evenly distributed in each compost pile. About 3 kg of samples were collected from each composting pile. The composting samples were stored in a refrigerator at 4 °C and immediately sent to the laboratory. The DOM extraction procedure was based on our previous studies, and the detailed extraction method is provided in Text S2. 

### 3.3. Fluorescence Spectroscopy

Prior to the DOM EEM spectra analysis, a SHIMADZU TOC-Vcph analyzer was used to determine the dissolved organic carbon concentration. All the DOM samples were diluted to 10 mg/mL to eliminate the inner-filter effect [20,21,22,23]. Detailed fluorescence analysis procedures are provided in Text S3.

### 3.4. Parallel Factor Analysis

The PARAFAC analysis was conducted based on the standard analysis procedure [24,38,39]. Furthermore, detailed analysis steps were offered in Text S3.

### 3.5. Two-Dimensional Correlation Spectra Analysis

To illustrate the dynamic of DOM molecules during composting, two-dimensional correlation spectra (2DCOS) analysis was carried out on the excitation loadings data of the PARAFAC molecules. 2D Shige software was employed to generate 2DCOS maps. The spectra data were analyzed in software based on the generalized 2DCOS theory reported by Noda [15]. In 2DCOS analysis, excitation loadings data of time-dependent composting was acquired, and 2DCOS was produced using the composting time as perturbation. As for hetero 2DCOS analysis, different excitation loadings data were used to conduct hetero 2DCOS analysis. Detailed 2DCOS analysis procedure is offered in Text S1.

## 4. Conclusions

In this study, a combination of multiple technologies was employed to characterize the dynamic of DOM molecules, which overcame the defect that could only present the overall changes of a DOM fluorescence component in the traditional analytical method. Five DOM PARAFAC components were found in the composting process. EEM/PARAFAC combined with 2DCOS can offer the interaction and dynamics information of the DOM peaks. In the composting process, the DOM fluorescence peaks in PARAFAC components presented the opposite trend. Simple structure DOM components could make a contribution to the formation of complex structure DOM components. Moreover, the interaction and dynamics information of the DOM molecules could be successfully characterized using EEM/PARAFAC combined with 2DCOS. 

## Figures and Tables

**Table 1:** DOM components’ spectral characteristics were identified by PARAFAC modeling for the EEM data set collected at different composting times. (Note: Naming rules for the fluorescence peaks in this study were: ① peak X corresponds to the peak in reference. ② Letter C and a number in brackets represent the fluorescence molecular number in this study.)

Fluorescent Components	Peak Position λEx/Em (nm)	Description and Probable Source	Reference λEx/Em (nm)
C1	220, 270/305	Tyrosine-like compounds characterized as fluorescence peak B1(C1) and peak B2(C1)	Peak B1 = 220–235/304–310 [20] Peak B2 = 270–280/304–310 [20] Component 4 = 274/306 [7] Component 4 = 260–290/290–340 [11]
C2	230, 280/340	Tryptophan-like compounds characterized as fluorescence peak T1(C2) and peak T2(C2)	Peak T1 = 220–235/334–360 [21] Peak T2 = 270–280/334–360 [21] Component 7 = 240, 300/338 [22] Component 3 = 220, 280/340 [11]
C3	245, 290, 320–360/410	Humic-like compounds characterized as fluorescence peak A1(C3), peak M1(C3), and peak M2(C3)	Peak A = 260/380–460; Peak M = 312/420–480 [21] Component 1 = 230, 330/410 [23] Component 8 = 250, 380/416 [22] Component 2 = <250, 305/412 [24]
C4	270, 370/460	A combination of humic-like peak A2(C4) and the ubiquitous humic-like peak C(C4)	Peak A = 260/380–460; Peak C = 350/420–480 [21] Component 3 = 270, 360/478 [7] Component 1 = 250–275(280–400)/370–500 nm [25] Component 2 = 240, 360/466 [11]
C5	220, 280, 330/405	Terrestrial humic-like substances; Biological; similar to the lawsone plotted in fluorescence spectra (peak L1(C5), peak L2(C5), and peak L2(C5) shoulder).	Component 3 = 295/398 [26] Component 2 = 315/418 [22] Component Q3 = 250–260/388 nm [27]

## Data Availability

Data available on request due to restrictions privacy.

## Supplementary Materials

The following supporting information can be downloaded at: https://www.mdpi.com/article/10.3390/molecules27217316/s1.
