# Supplementary material for: Revealing the Inner Changes of Component Composition Derived from DOM PARAFAC Based on Two-Dimensional Correlation Spectroscopy
Source: Molecules. 2022 Oct 27;27(21):7316. doi: 10.3390/molecules27217316 (PMC9655640; doi:10.3390/molecules27217316)
Supplement: Supplementary file 1 [file molecules-27-07316-s001.zip › molecules-1931400-supplementary.pdf]

Revealing the Inner Changes of Components Composition Derived from DOM PARAFAC Based on Two-Dimensional Cor-relation Spectroscopy

Hongyang Cui[1,2,3], Lina Xie[1], Guogang Zhang[1], Yue Zhao[2], Zimin Wei[1,2*]

[1] Tianjin Key Laboratory of Animal and Plant Resistance, College of Life Sciences, Tianjin Normal University, Tianjin 300387, China

[2] College of Life Science, Northeast Agricultural University, Heilongjiang 150030, People's Republic of China

[3] *Laboratory for Earth Surface Processes,* College of Urban and Environmental Sciences, Peking University, Beijing 100871, China

[*]Address for Correspondence

Prof. Zimin Wei

Tianjin Normal University

E-mail address: weizimin@neau.edu.cn or weizm691120@163.com

This supporting information contains 19-page document including text, 5 figures, 2 tables, and a reference list.

Text S1.

The DOM was immediately extracted, and all measurements were made in the laboratory within 2 days after collection. Extraction of DOM from the composting samples was performed in a horizontal shaker with Milli-Q water (solid-to-water ratio: 1:10, w/v) for 24 h at room temperature. The suspensions were centrifuged at 10000 rpm for 10 min and filtered through 0.45-μm polycarbonate filters. Before filtering, the polycarbonate filters were cleaned with Milli-Q water.

Text S2.

The pH value of the DOM samples was adjusted to the desired level (pH=7.0±0.05) by adding 0.10 M HCl or NaOH solution. The fluorescence instrument was corrected according to the manufacturer instructions before analyzing the samples. To eliminate the inner-filter effect, the EEMs were corrected using the measured absorption spectra. EEMs spectra were determined for all composting samples, and Milli-Q (Millipore) water with a Hitachi F-7000 fluorescence spectrophotometer (Hitachi High Technologies, Japan) in a 1-cm clear quartz cuvette at room temperature (20±2 °C). The emission wavelength over the range 250-600nm was collected in 2-nm increments, and the excitation wavelength gradually increased from 200 to 550 nm in 1-nm increments. The scan speed was set at 2400nm min^{-1}. In addition, the fluorescence spectrum of Milli-Q water was subtracted from the spectra of the composting DOM to eliminate the influence of Rayleigh and Raman scattering.

The PARAFAC was employed to investigate the EEMs spectral data at each sampling time. The number of samples included in the PARAFAC model at each sampling time was 42 (7 composting piles × 6 samples). The PARAFAC analysis was carried out in MATLAB 2013a (Mathworks, Natick, MA) with the DOMFluor toolbox (www.models.life.ku.dk), following the procedure described by Stedmon and Bro.[38] Several processing steps were used to minimize the influence of scatter lines and other attributes of the EEMs landscape. The EEMs of a control Milli-Q water was subtracted from each EEMs from the studied samples to remove the lower-intensity Raman scatters lines. The Rayleigh and Raman scatters were corrected by the protocol of Bahram et al..[25] The

concentration scores of the PARAFAC components were expressed as the maximum fluorescence intensity ($F_{\max}$) for each modeled component. F_{max} were reported in Raman Units (R.U.) in this study.[26]

In this study, the results of explained variance, core consistency, split-half, residuals analysis and visual inspection were employed to determine the appropriate components.[3-5] 42 EEMs (7 composting piles × 6 samples) were included in each PARAFAC model at each sampling time. The results of explained variance and core consistency of PARAFAC analysis at each composting time are presented in Table S1. The explained variance indicates that four components are optimal because the increase obtained with more than four components is small relative to the increase in explained variance obtained using up to four components (Table S1). Additionally, the percentage of variance explained using four components is adequate for these types of data. The core consistency diagnostic is an approach suggested for finding the number of components in PARAFAC analysis. If the core consistency is not close to 100%, the model does not give an appropriate description of the data and a lower number of components should be chosen. Core consistencies for two to six components models are shown in Table S1. The core consistencies for two to four components models were all close to 100%, but the core consistency for five to six components models were significantly lower than 100%. This indicated that five to six component-models might not be stable. Based on the result of split-half analysis, in different sampling time, four components model were considered to satisfy the validation in three independent data sets to ensure the correctness of the modeled DOM components by the PARAFAC model using the split validation procedure (Figure S1). Furthermore, the sum of squared residuals in the excitation and emission directions of different sampling time was plotted in Figure S2. It was clear that more than three components were suitable for the datasets of different composting time. Combining with the results of explained variance, core consistency, split-half analysis, residuals analysis and visual inspection, the validation results showed that four components were appropriate (Table S1, Figure S1, S2 and S3). However, the five components model could not satisfy the validation. Therefore, the fluorescence EEMs of different sampling time of composting materials could be successfully decomposed into four-component model by PARAFAC.

Text S3.

Component C1 was characterized by two fluorescence peaks, and its maximum excitation/emission (Ex/Em)

wavelength pair was centered at 220 (270)/305 nm (Figure 1, Table 1). These fluorescence characteristics could be categorized as the previously defined tyrosine-like component peak B1(C1) and peak B2(C1).[6, 7] The spectral features are similar to other reported protein-like component from landfill leachate and sewage.[8, 9] Component C2 was also composed of two peaks with excitation maxima at 230 and 280 nm, and emission maxima was observed in the EEMs at 340 nm (Figure 1, Table 1). This component was characterized as tryptophan-like component peak T1(C2) and peak T2(C2).[6, 7]

Component C3 showed fluorescence peaks at excitation/emission wavelength pair of 245, 290, (320-360)/410 nm (Figure 1, Table 1), which resembled a combination of previously defined humic-like fluorescence components peak A1(C3), peak M1(C3) and peak M2(C3).[6, 10] Component C4 showed excitation peaks at 270 and 370 nm, with an emission maximum at 460 nm (Figure 1, Table 1). This component was characterized as a combination of terrestrial humic-like peak A2(C4) and the ubiquitous humic-like peak C(C4) according to Coble.[21] Fluorescence component C3 and C4 were similar to microbial oxidized components and humic components produced through biogeochemical processing.[11, 12]

As composting time increase, the component C5 formed by microbial activity and observed by fluorescence EEM spectra. Component C5 of 35d (Figure 1, Table 1) was composed of two excitation maxima at 220 and 280 nm and one emission peak at 405 nm which has been identified as dissolved organic matter that has been altered by microbial reprocessing.[13] In addition, the peak at shorter excitation wavelength (220nm) of component C5 appeared at 35d was often considered the microbial humic fluorescence at the UVA range.[14] However, component C5 of 45 and 60d exhibited excitation maxima at 280 and emission maxima at 405 nm, with the absence of excitation maxima peak at 220nm. Component C5 of 45 and 60d was reported to be related to microbial formed fulvic acid-like substance by a long aliphatic chain.[9, 21]

Text S4.

Figure S4 a, b shows the synchronous and asynchronous maps of the hetero-2DCOS analyzed by excitation loadings of component C1 and C2. Remarkably, in synchronous hetero-2DCOS, peak B1(C1) was

negative correlated to the peak T1(C2) but positive correlated to peak T2(C2) (Figure S4 a; Table S3). Peak B2(C2) was negative correlated to peak T1(C2), and peak B2(C2) was not detected correlational relationship with peak T2(C2). These results indicated that the tyrosine-like component peak B1(C1) was the basic fluorescent units for tryptophan-like component peak T2(C2), and peak B2(C1) had no relationship with component C2 during their evolution. In the asynchronous map (Figure S4 b; Table S3), there were two negative signs at coordinates (220nm, 280nm) and (270nm, 280nm) and two positive signs at (220nm, 230nm) and (270nm, 230nm), indicating that the fluorescence response of tyrosine-like component occurred before the spectral changes of tryptophan-like component peak T1(C2), but after the spectral changes of tryptophan-like component peak T2(C2).

The synchronous and asynchronous maps of the hetero-2DCOS analyzed by excitation loadings of PARAFAC component C1 and C3 are presented in fig. S4 c, d. In synchronous hetero-2DCOS, peak B1(C1) and peak B2(C1) were all negative correlated to peak M1(C3) but positive correlated to peak M2(C3) (Figure S4 c and d; Table S3), indicating that tyrosine-like component peak B1(C1) and peak B2(C1) were the basic fluorescent units for humic-like component peak M2(C3). In asynchronous map fluorescence component C1 was positive correlated to peak M2(C3) but negative correlated to peak M1(C3), indicating that the fluorescence response of tyrosine-like component occurred before the spectral changes of humic-like component peak M2(C3), but after the spectral changes of humic-like component peak M1(C3).

Figure S4 e, f showed synchronous and asynchronous hetero-2DCOS C1/C4 excitation loadings correlation analysis. In synchronous hetero-2DCOS, fluorescence component C1 was positive correlated to peak C(C4) but negative correlated to peak A2(C4). In asynchronous hetero-2DCOS map, fluorescence component C1 was positive correlated to peak A2(C4) but negative correlated to peak C(C4) (Figure S4 e and f; Table S3). These results indicated that tyrosine-like component was the basic fluorescent units for humic-like component peak C(C4). The fluorescence response of tyrosine-like component occurred before the spectral changes of humic-like component peak A2(C4), but after the spectral changes of humic-like component peak

C(C4).

The dynamic relationships between fluorescence component C1 and C5 were analyzed by hetero-2DCOS (FigureS4 g, h; Table S3). From the synchronous hetero-2DCOS, we could learn that the fluorescence component C1 made a contribution to the formation of component C5 peak L2(C5) during composting. The asynchronous hetero-2DCOS map showed that the changing sequence of peaks in component C1 and C5 during composting followed the order peak L2(C5)→component C1→peak L1(C5).

Figure S4 i, j displayed the synchronous and asynchronous maps of the hetero-2DCOS analyzed by excitation loadings of component C2 and C3. Generally, there were four main cross-peaks and two shoulder peaks in the synchronous maps. Peak T1(C2) was negative correlated to peak M1(C3) but positive correlated to peak M2(C3). Peak T2(C2) was positive to peak M1(C3) but negative correlated to peak M2(C3). In asynchronous hetero-2DCOS map, peak T1(C2) was negative correlated to peak M1(C3) but positive correlated to peak M2(C3). Peak T2(C2) was positive to peak M1(C3) but negative correlated to peak M2(C3). These results revealed that the formation of peak M2 arise from the degradation of tryptophan-like component peak T1(C2), and peak T2(C2) arise from the humic-like component peak M1(C3) during composting. The fluorescence response of tryptophan-like component peak T1(C2) occurred before the spectral changes of humic-like component peak M2(C3), but after the spectral changes of humic-like component peak M1(C3). But the fluorescence response of tryptophan-like component peak T2(C2) occurred in an opposite sequence as peak T1(C2) with fluorescence peak M1(C3) and M2(C3).

The synchronous and asynchronous hetero-2DCOS maps analyzed by excitation loadings of PARAFAC component C2 and C4 are presented in fig. S4 k, l. In synchronous hetero-2DCOS map, fluorescence peak T1(C2) was positive correlated to peak C(C4) but negative correlated to peak A2(C4). Peak T2(C2) was positive correlated to peak A2(C4) but negative correlated to peak C(C4). In asynchronous hetero-2DCOS map, the cross-peaks presented similar peaks as synchronous hetero-2DCOS map. These results revealed that peak T1(C2) of tryptophan-like component was the basic fluorescent units for humic-like component peak C(C4) and

humic-like peak A2(C4) the basic fluorescent units for tryptophan -like component peak T2(C2) during composting. The fluorescence response of peak T1(C2) occurred after the spectral changes of peak A2(C4), but before the spectral changes of peak C(C4). The fluorescence response of peak T2(C2) occurred in an opposite sequence as T1(C2) with fluorescence peak A2(C4) and C(C4).

Figure S4 m, n showed the synchronous and asynchronous hetero-2DCOS spectra analyzed by the excitation loadings of C2 and C5. The peaks in synchronous map indicated that the decomposition of peak T1(C2) made a contribution to the formation of peak L1(C5) during composting. And the peak T2(C2) and L2(C5) had the same origin. The signs of cross-peaks in the asynchronous map indicated that the sequence of the C2 and C5 peaks follows the order peak T1(C2)→L1(C5)→T2(C2)→L2(C5).

Figure S4 o, p showed the synchronous and asynchronous maps of the hetero-2DCOS maps analyzed by excitation loadings of component C3 and C4. In synchronous hetero-2DCOS map, fluorescence peak M1(C3) was positive correlated to peak A2(C4) but negative correlated to peak C(C4). Peak M2(C3) was positive correlated to peak C(C4) but negative correlated to peak A2(C4). In asynchronous hetero-2DCOS map, the cross-peaks of C3/C4 presented similar peaks as synchronous hetero-2DCOS map. These results indicated that dynamics of peak M1(C3) was similar to that of peak A2(C4). And the peak M2(C3) and peak C(C4) had a same origin during composting. The fluorescence response of peak M1(C3) occurred before the spectral changes of peak A2(C4), but after the spectral changes of humic-like component peak C(C4). The fluorescence response of peak M2(C3) occurred in an opposite direction as M1(C3) with fluorescence peak A2(C4) and C(C4).

The dynamic relationships between fluorescence component C3 and C5 were analyzed by hetero-2DCOS (FigureS4 q, r). The cross-peaks in synchronous hetero-2DCOS map showed that peak M1(C3) was transformed into peak L1(C5) during composting. And the peak M2(C3) and L2(C5) had a same origin. In asynchronous hetero-2DCOS map, the cross-peaks showed that changing sequence of peaks in component C3 and C5 during composting followed the order peak M1(C3)→L2(C5)→M2(C3)→L1(C5).

Figure S4 s, t showed the synchronous and asynchronous hetero-2DCOS maps analyzed by excitation loadings of component C4 and C5. In synchronous hetero-2DCOS map, the peaks indicated that peak A2(C4) was the basic fluorescent units for peak L2(C5). Peak C(C4) and peak L1(C5) had a same origin during composting. The cross-peaks in asynchronous map showed that changing sequence of peaks in component C4 and C5 during composting followed the order peak A2(C4)→L1(C5)→C(C4)→L2(C5).

Text S5.

To obtain the structural variation information on DOM PARAFAC components, 2DCOS was employed using the excitation loadings of PARAFAC components with composting time as the external perturbation. The 2DCOS spectra were produced based on the method of Noda and Ozaki.[15] For the spectral changes of $y(v, t)$ as a function of a spectral variable (v) and an external variable (t, composting time).

And the dynamic spectrum $\tilde{y}(v, \mathrm{t})$ is defined as follows:

$$\tilde{y}(v,\mathrm{t}) = \begin{cases} y(v,\mathrm{t}) - \bar{y}(v) & \text{for } \mathrm{T_{min}} \le \mathrm{t} \le \mathrm{T_{max}} \\ 0 & \text{otherwise} \end{cases} \quad (1)$$

where $\bar{y}(v)$ is the reference spectrum of the system.[15] For the reference spectrum it is customary to set $\bar{y}(v)$ as the stationary or average spectrum given by

$$\bar{y}(v)=\frac{1}{\mathrm{T}}\int_{-\frac{\mathrm{T}}{2}}^{\frac{\mathrm{T}}{2}} y(v,t)\mathrm{d}t \quad (2)$$

A computational method according to the discrete Hibert-Noda transform is employed to generate a pair of correlation intensity maps, i.e., synchronous (Φ) and asynchronous (Ψ) 2DCOS.

A 2D synchronous spectrum is given by

$$\Phi(v_1,v_2)=\frac{1}{\mathrm{T_{max}}-\mathrm{T_{min}}}\int_{\mathrm{T}}^{\mathrm{T_{max}}} \tilde{y}(v_1,\mathrm{t}) \times \tilde{y}(v_2,\mathrm{t})\mathrm{dt} \quad (3)$$

The 2D asynchronous spectrum can be calculated from the cross-correlation of the dynamic spectrum, and its orthogonal spectrum $\tilde{z}(v,\mathrm{t})$. The mathematical expression is given by

$$\Psi(v_1,v_2)=\frac{1}{\mathrm{T_{max}}-\mathrm{T_{min}}}\int_{\mathrm{T}}^{\mathrm{T_{max}}} \tilde{y}(v_1,\mathrm{t}) \times \tilde{z}(v_2,\mathrm{t})\mathrm{dt} \quad (4)$$

The intensity of a synchronous 2DCOS represents the simultaneous or coincidental changes of two separate spectral intensity variations measured at v_1 and v_2 during the interval between $\mathrm{T_{min}}$ and $\mathrm{T_{max}}$ of the externally defined variable t (which is not limited to time as external disturbance, but suitable for concentration, pH, and temperature and so on.). The intensity of peaks located at diagonal positions mathematically corresponds to the autocorrelation function of spectral intensity variations observed during an interval between

T_{min} and T_{max}. The diagonal peaks are therefore referred to as autopeaks, and the magnitude of an autopeak intensity, which is always positive, represents the overall extent of spectral intensity variation observed at the specific spectral variable *v* during the observation interval between T_{min} and T_{max}. Thus, an autopeak represents the overall susceptibility of the corresponding spectral region to change in spectral intensity as an external perturbation is applied to the system. Cross peaks located at the off-diagonal positions of a synchronous 2DCOS represent simultaneous or coincidental changes of spectral intensities observed at two different spectral variables v_1 and v_2. Such a synchronized change suggests the possible existence of a coupled or related origin of the spectral intensity variations. The sign of synchronous cross peaks is either increasing or decreasing together as function of the external variable t during the observation interval. However, the negative sign of cross peaks suggests that one to the spectral intensities is increasing while the other is decreasing.

Asynchronous 2DCOS, the imaginary part of the cross-correlation function, show cross-peaks exclusively. Their signs reveal the sequential order of the dynamics of spectral intensity variations induced by the perturbation. The same signs of synchronous and asynchronous cross-peaks indicate that the spectral intensity change at v_1 occurs predominantly before that of v_2 along the perturbation variable axis. If the signs of synchronous and asynchronous cross-peaks are different, the intensity change at v_1 occurs after v_2.

2DCOS was applied to the excitation loadings of PARAFAC components. 2DCOS spectra were calculated by using the algorithm according to the numerical method developed by Noda, 2DCOS and hetero-2DCOS maps were obtained using 2D Shige software (Kwansei-Gakuin University, Japan). 2DCOS and hetero-2DCOS maps were plotted using Origin 2015 software.

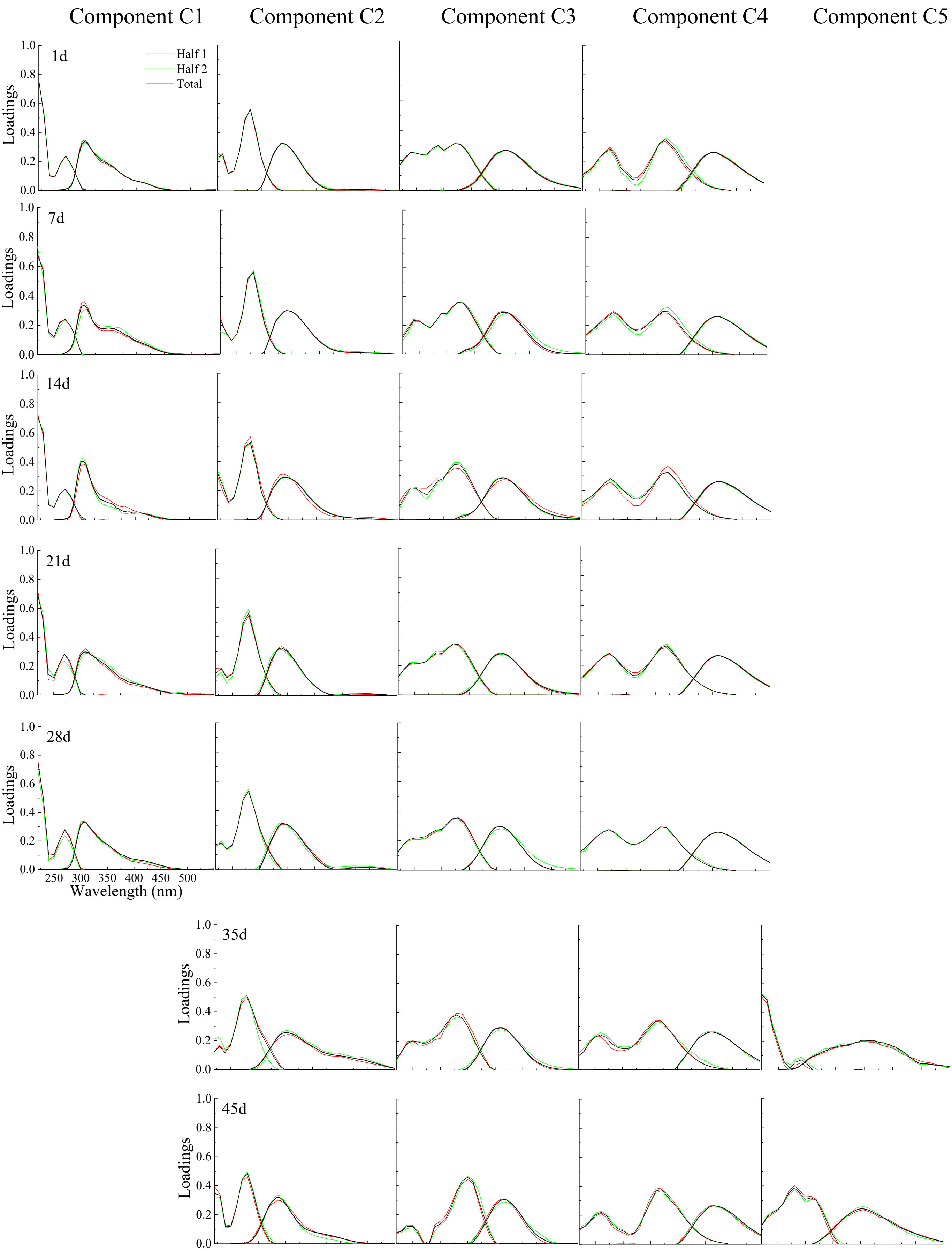

Component C1
Component C2
Component C3
Component C4
Component C5
Half 1
Half 2
Total
1d
7d
14d
21d
28d
35d
45d
Loadings
Wavelength (nm)
0.0
0.2
0.4
0.6
0.8
1.0
250
300
350
400
450
500

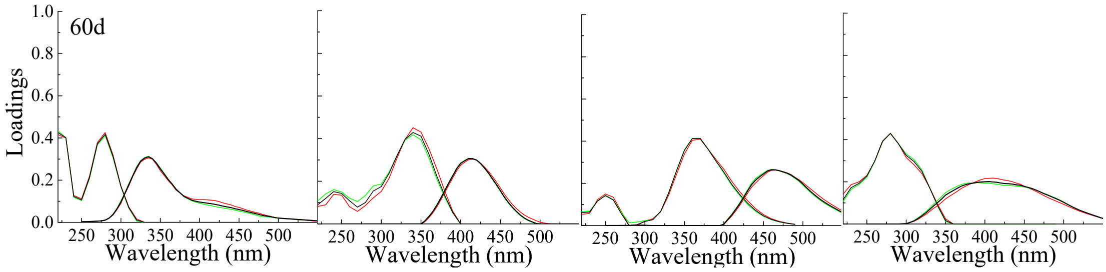

Figure S1. Spectral loadings of split-half validation results of the components in the composting of different sampling time; excitation (left) and emission (right) spectra were estimated from the two independent halves of the dataset (red and green lines) and the complete dataset (black lines).

0d

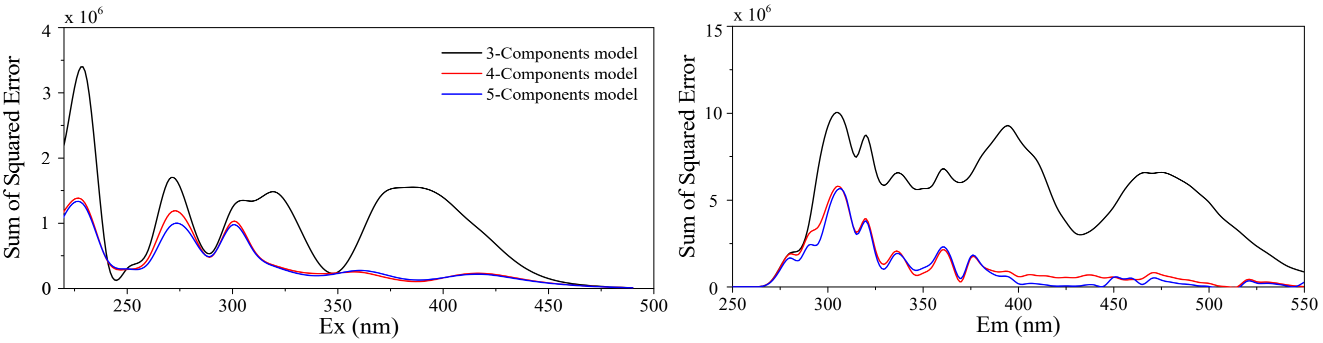

7d

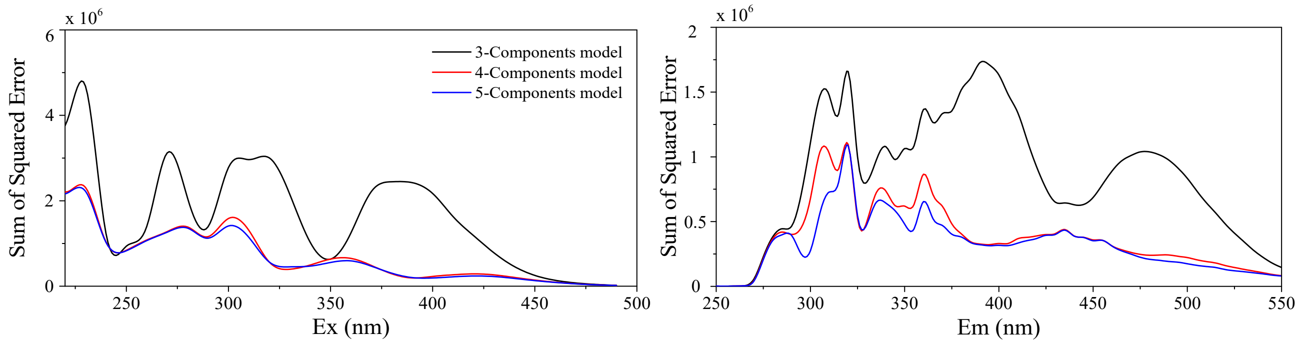

14d

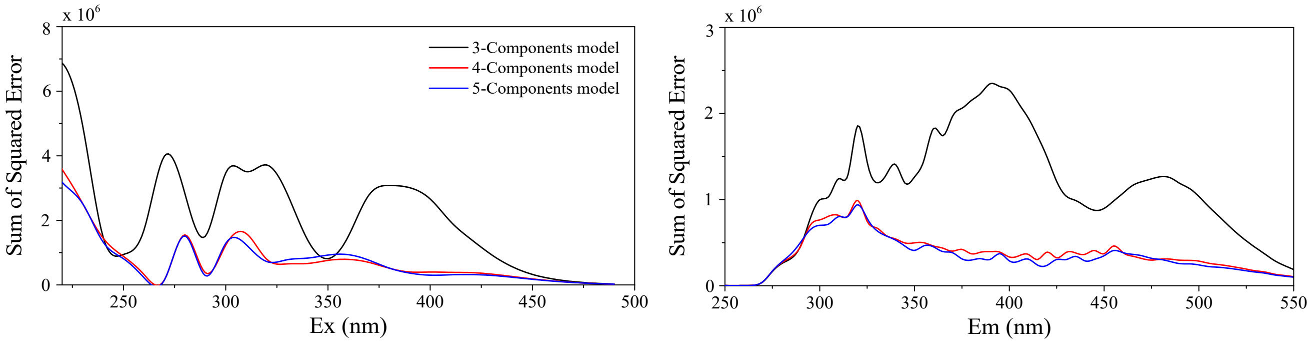

21d

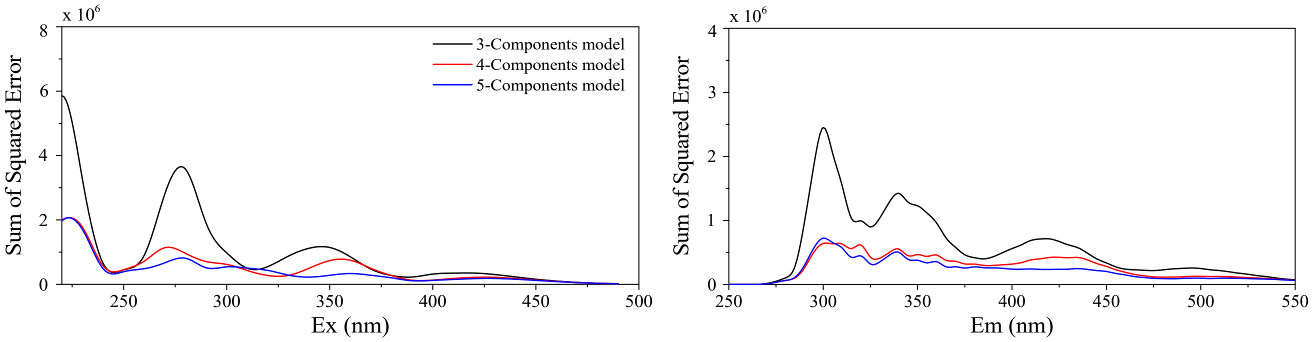

28d

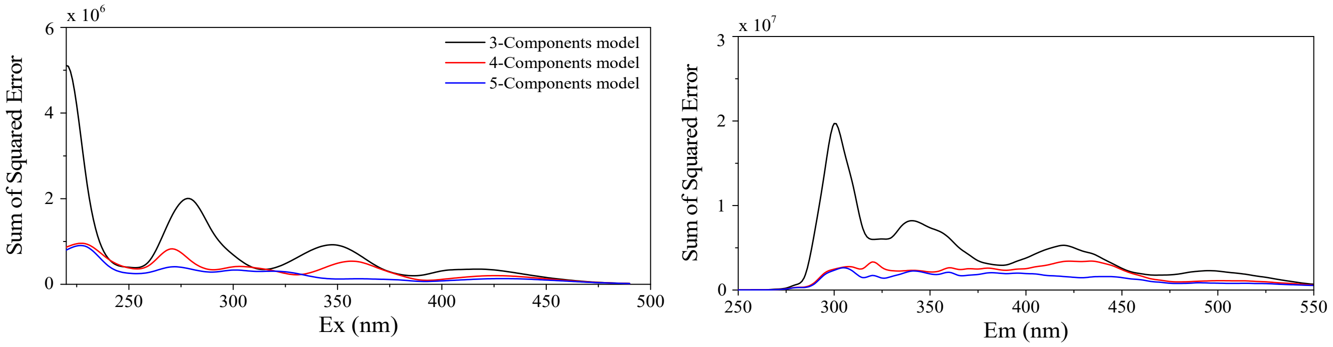

35d

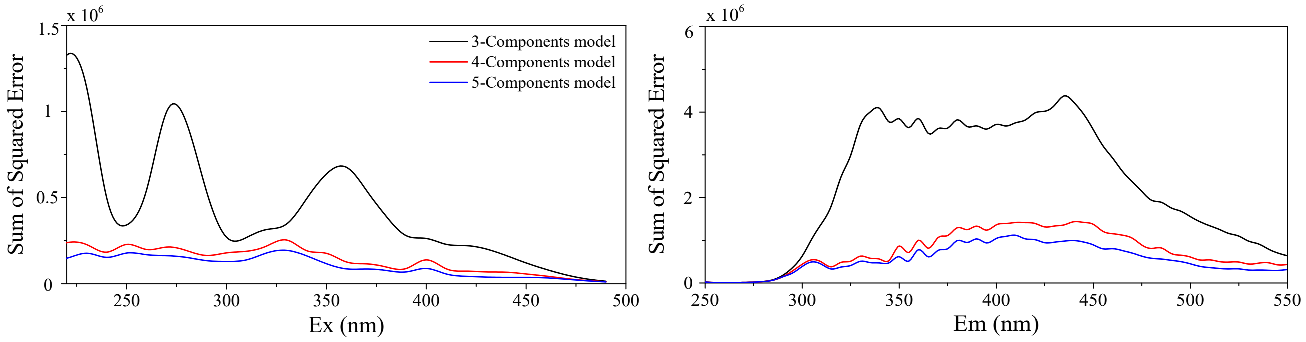

45d

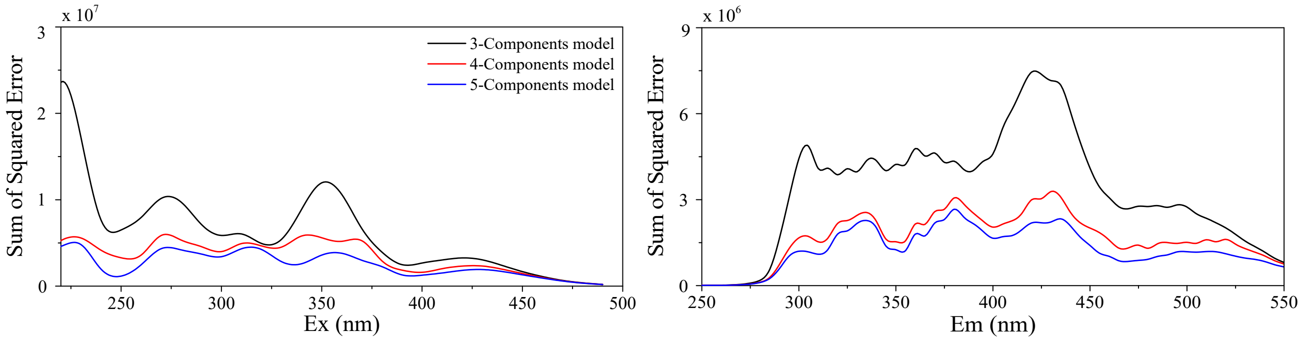

60d

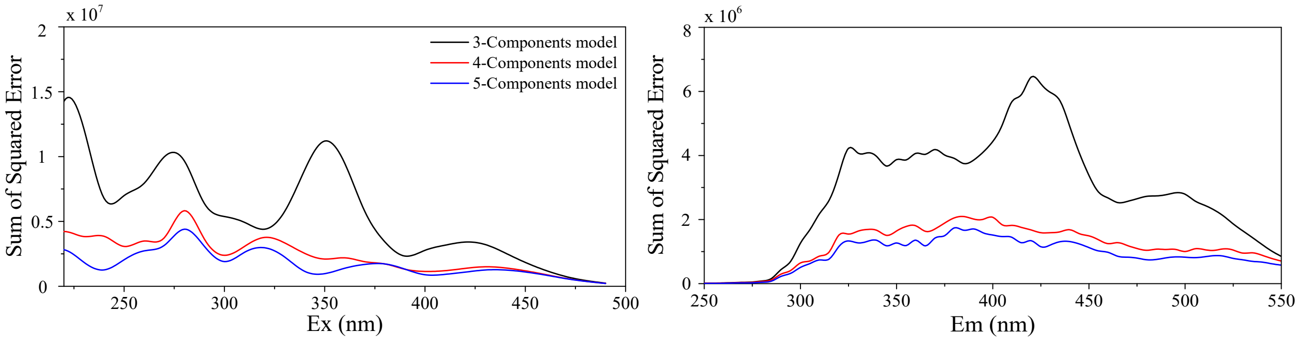

Figure S2. Sum of squared error of the different numbers of PARAFAC components in different sampling time for determining the components numbers.

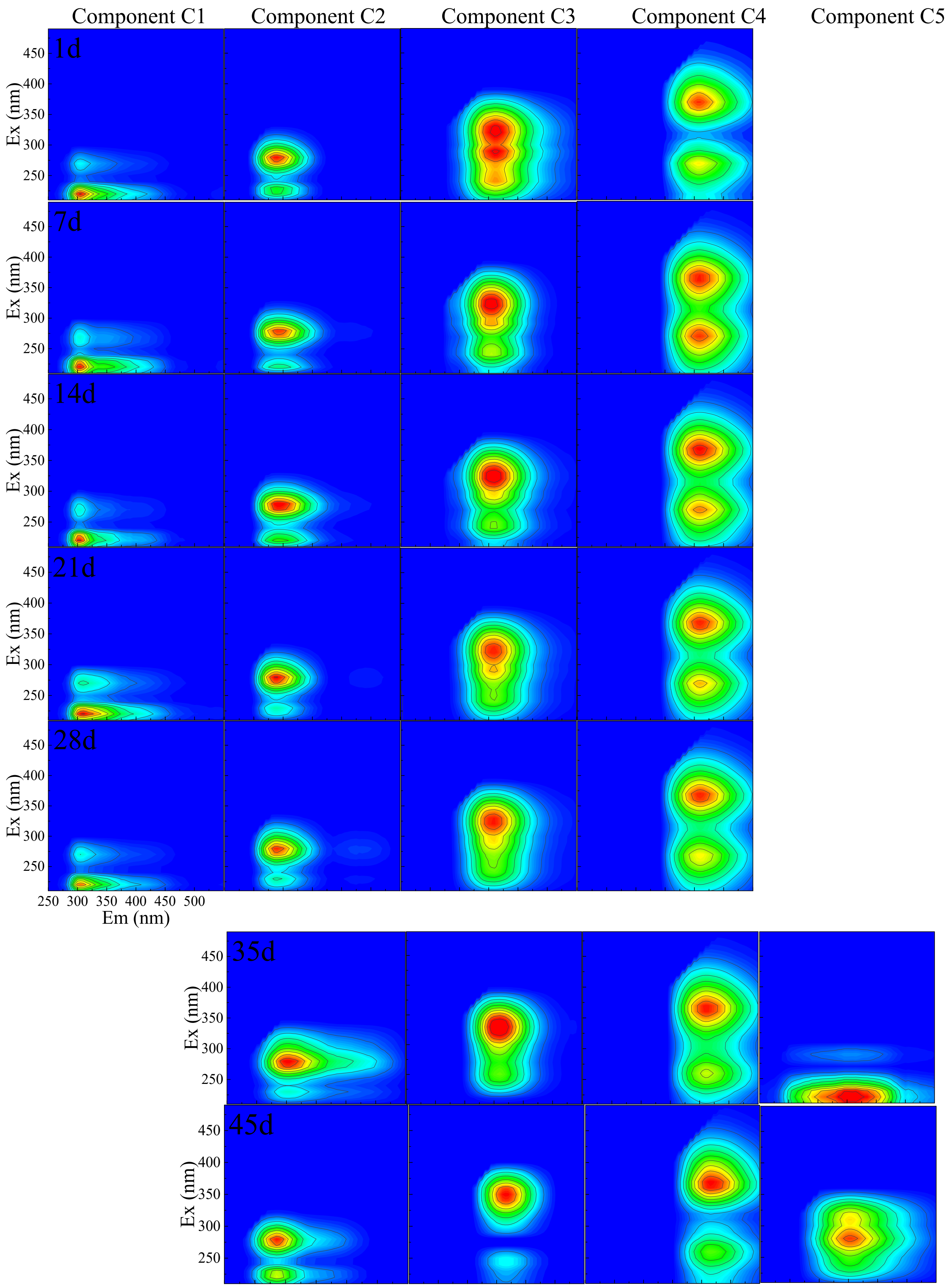
Component C1
Component C2
Component C3
Component C4
Component C5
1d
7d
14d
21d
28d
35d
45d
Ex (nm)
450
400
350
300
250
Em (nm)
250 300 350 400 450 500

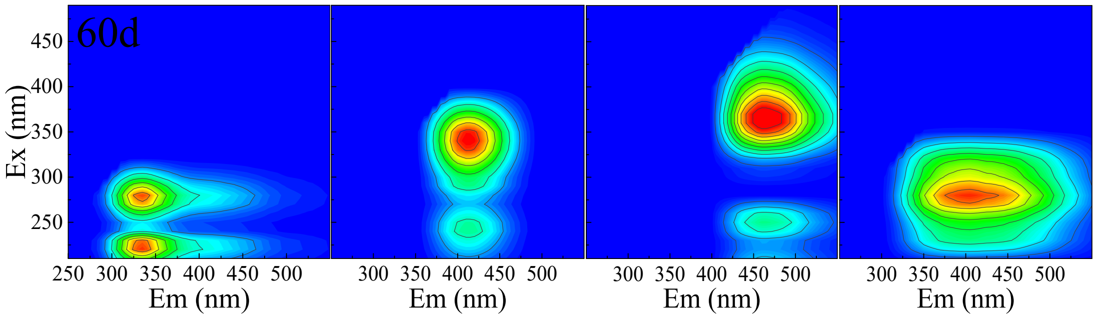

Figure S3. The different fluorescent components found by the PARAFAC model in each sampling time.

Refer to Figure1 and Table 1 for the positions of their maxima.

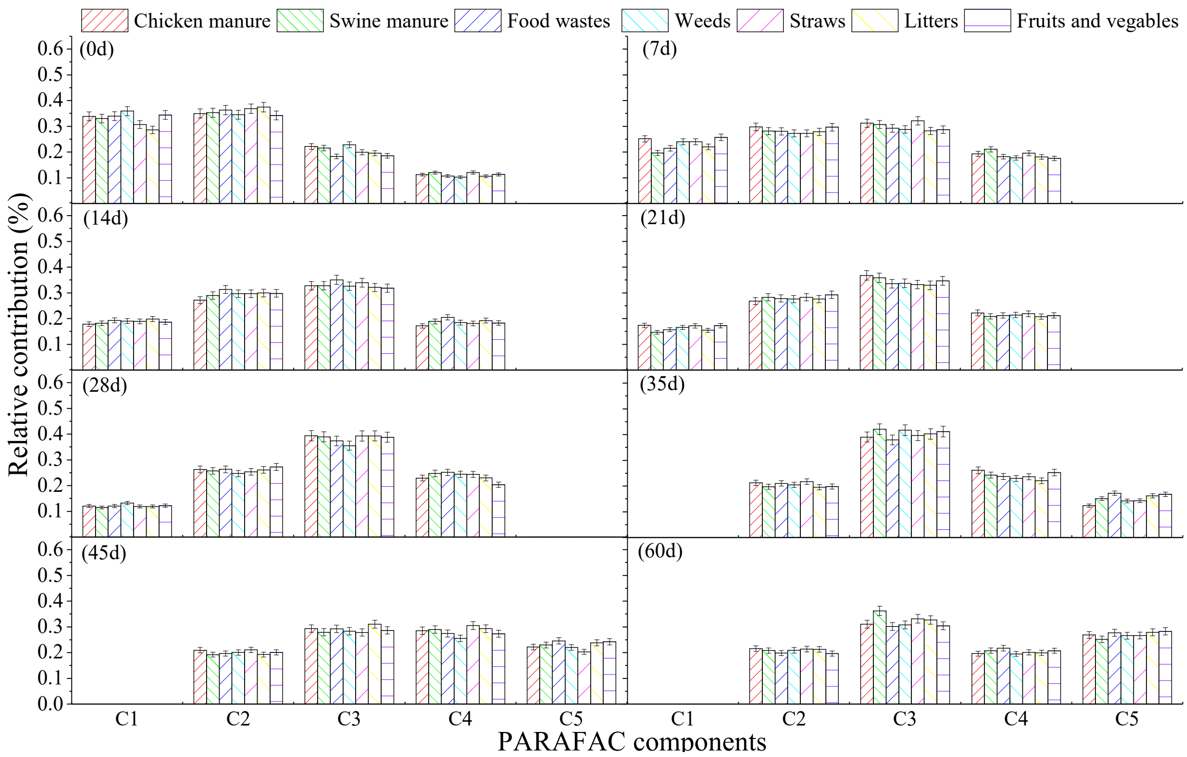

Figure S4. The relative contributions (F_{max} %) of the PARAFAC-modeled components in each compost pile during composting.

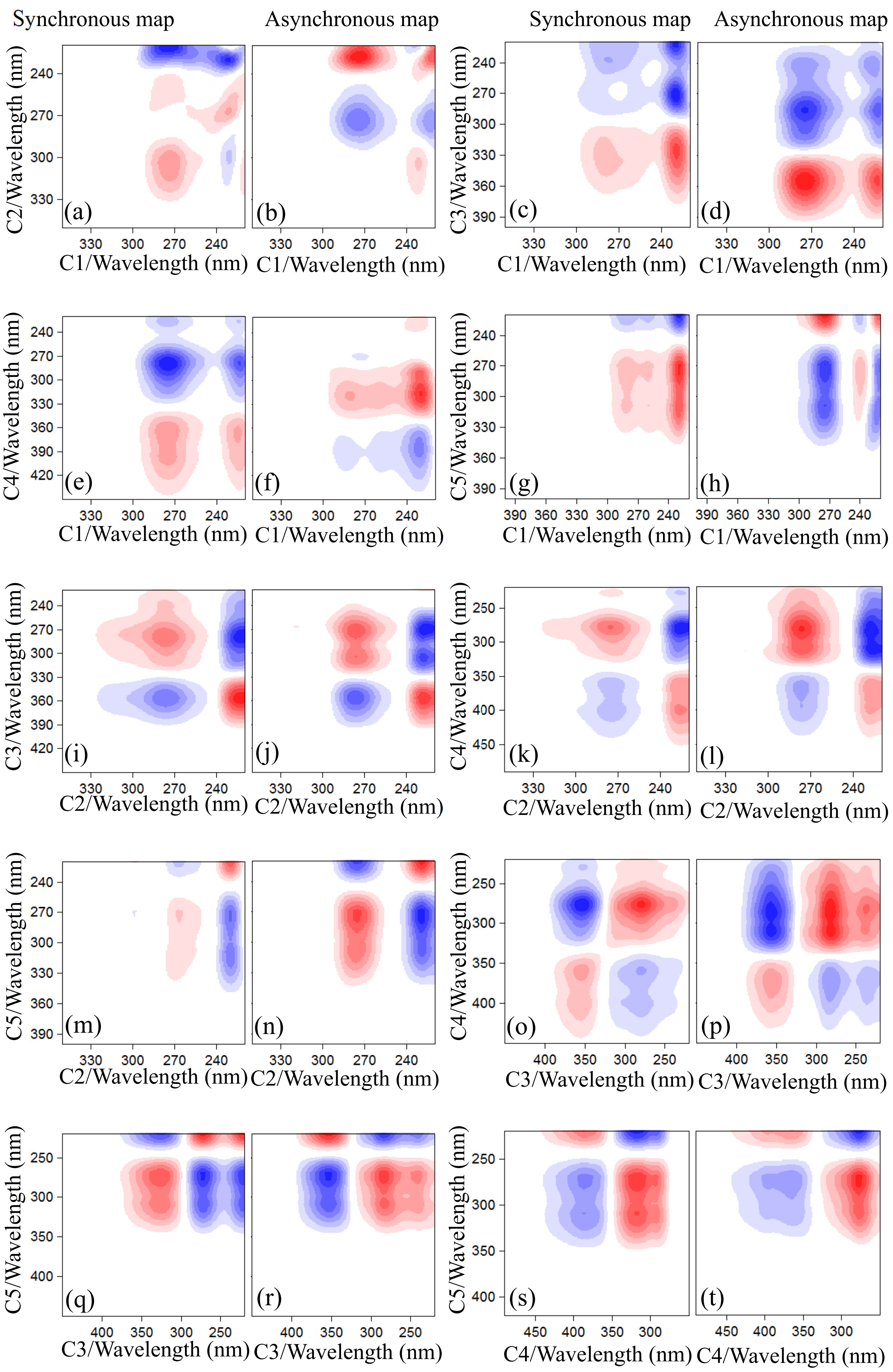

Figure S5 Synchronous and asynchronous hetero-spectral two-dimensional correlation spectrum. *Red* and *blue* represents positive and negative cross peaks.

Table S1. Explained variance and core consistency as a percentage vs the number of components for PARAFAC models of the fluorescence data with 2-6 components.

		No. of components				
		2	3	4	5	6
1d	Explained variance (%)	96.3	98.0	99.1	99.2	99.5
	Core consistency (%)	99.8	98.9	98.5	13.9	13.0
7d	Explained variance (%)	96.7	98.5	99.5	99.7	99.8
	Core consistency (%)	99.9	97.8	96.8	32.3	13.3
14d	Explained variance (%)	95.8	97.8	99.0	99.3	99.5
	Core consistency (%)	100.0	98.9	98.7	37.9	18.5
21d	Explained variance (%)	96.0	98.1	99.2	99.4	99.6
	Core consistency (%)	99.8	99.8	98.9	31.6	28.8
28d	Explained variance (%)	97.2	98.1	99.6	99.6	99.8
	Core consistency (%)	99.7	98.9	98.3	41.4	9.8
35d	Explained variance (%)	97.7	98.2	99.5	99.8	99.9
	Core consistency (%)	99.9	99.2	99.0	27.0	20.1
45d	Explained variance (%)	97.1	98.0	99.4	99.7	99.8
	Core consistency (%)	99.8	99.3	98.8	39.5	8.6
60d	Explained variance (%)	96.7	98.7	99.5	99.6	99.8
	Core consistency (%)	99.6	98.9	98.5	28.7	10.2

Table S2. Fluorescence intensities changes of the peaks in PARAFAC components during composting.

	C1		C2		C3			C4		C5	
	Peak B1	Peak B2	Peak T1	Peak T2	Peak A1	Peak M1	Peak M2	Peak A2	Peak C	Peak L1	Peak L2
1d	0.204	0.053	0.088	0.177	0.061	0.082	0.083	0.077	0.094	-	-
7d	0.197	0.052	0.081	0.179	0.061	0.080	0.104	0.076	0.097	-	-
14d	0.194	0.058	0.086	0.175	0.067	0.076	0.106	0.073	0.096	-	-
21d	0.183	0.046	0.069	0.181	0.067	0.081	0.108	0.073	0.097	-	-
28d	0.123	0.045	0.063	0.183	0.071	0.076	0.117	0.078	0.099	-	-
35d	-	-	0.041	0.186	0.079	0.066	0.117	0.083	0.109	0.108	0.014
45d	-	-	0.027	0.196	0.063	0.022	0.139	0.068	0.119	0.093	0.077
60d	-	-	0.032	0.178	0.074	0.031	0.137	0.037	0.129	0.023	0.086
